# Cortical Layer and Spectrotemporal Architecture of Epileptiform Activity *in vivo* in a Mouse Model of Focal Cortical Malformation

**DOI:** 10.3389/fncir.2019.00002

**Published:** 2019-01-22

**Authors:** Anthony J. Williams, Qian-Quan Sun

**Affiliations:** Department of Zoology & Physiology, University of Wyoming, Laramie, WY, United States

**Keywords:** high frequency oscillations, epilepsy, spike wave discharge, single unit, neonatal freeze lesion

## Abstract

Our objective is to examine the layer and spectrotemporal architecture and laminar distribution of high-frequency oscillations (HFOs) in a neonatal freeze lesion model of focal cortical dysplasia (FCD) associated with a high prevalence of spontaneous spike-wave discharges (SWDs). Electrophysiological recording of local field potentials (LFPs) in control and freeze lesion animals were obtained with linear micro-electrode arrays to detect presence of HFOs as compared to changes in spectral power, signal coherence, and single-unit distributions during “hyper-excitable” epochs of anesthesia-induced burst-suppression (B-S). Result were compared to HFOs observed during spontaneous SWDs in animals during sleep. Micro-electrode array recordings from the malformed cortex indicated significant increases in the presence of HFOs above 100 Hz and associated increases in spectral power and altered LFP coherence of recorded signals across cortical lamina of freeze-lesioned animals with spontaneous bursts of high-frequency activity, confined predominately to granular and supragranular layers. Spike sorting of well-isolated single-units recorded from freeze-lesioned cortex indicated an increase in putative excitatory cell activity in the outer cortical layers that showed only a weak association with HFOs while deeper inhibitory units were strongly phase-locked to high-frequency ripple (HFR) oscillations (300–800 Hz). Both SWDs and B-S show increases in HFR activity that were phase-locked to the high-frequency spike pattern occurring at the trough of low frequency oscillations. The spontaneous cyclic spiking of cortical inhibitory cells appears to be the driving substrate behind the HFO patterns associated with SWDs and a hyperexcitable supragranular layer near the malformed cortex may play a key role in epileptogenesis in our model. These data, derived from a mouse model with a distinct focal cortical malformation, support recent clinical data that HFOs, particularly fast ripples, is a biomarker to help define the cortical seizure zone, and provide limited insights toward understanding cellular level changes underlying the HFOs.

## Introduction

Focal epilepsy is associated with the derangement of local neural network activity leading to hypersynchronous neuronal discharges (ictogenesis) in the affected brain tissues (van Diessen et al., 2013; Perucca et al., 2014; Ferrari-Marinho et al., 2015). High frequency oscillations (HFOs) have recently emerged as a sensitive biomarker of hyper-excitable tissues responsible for focal epilepsy (Brázdil et al., 2010; Ferrari-Marinho et al., 2015; van Klink et al., 2016; Cuello-Oderiz et al., 2018) and resection of brain regions with increased HFO rates has been associated with a high incidence of seizure-free outcome in clinical patients (Fujiwara et al., 2012; Fedele et al., 2016; Cuello-Oderiz et al., 2018). However, both physiological and pathological HFOs are common within cortical brain regions of epileptic patients (Matsumoto et al., 2013) though fast ripple activity (>250 Hz), Currently however, the spectral characteristics that distinguish physiological vs. pathological HFOs have not been clearly defined (Kerber et al., 2013; Frauscher et al., 2015).

Focal cortical dysplasia (FCD) is a common form of cortical malformation associated with a high incidence of epilepsy (Guerrini and Dobyns, 2014; Luhmann, 2016). Given the focal nature of FCD, many of these patients exhibiting refractory epilepsy are candidates for surgical resection of the affected brain tissue to relieve the epileptic condition (Sisodiya, 2000). The hyperexcitability that arises within the SOZ likely involves the accumulation of hypersynchronous unit activity that ultimately interferes with normal brain rhythms and can broadcast pathological activities to adjacent brain regions through low frequency propagation patterns (e.g., spike-and-wave seizure discharges). Recent evidence suggests that specific ictal patterns may indicate distinct network alterations driving epileptogenesis as demonstrated in drug-resistant epilepsy patients (Ogren et al., 2009; Ferrari-Marinho et al., 2016) and in animal models (Bragin et al., 2009; Lévesque et al., 2012). Distinct differences in network connectivity has also been demonstrated between different types of cortical malformations as demonstrated from cellular synaptic activity of epileptic tissues obtained from clinical patients (Cepeda et al., 2012). The spectrotemporal dissection of pathological waveforms obtained with high-density micro-electrode arrays including the analysis of cross-laminar and cross-frequency coupling may serve as useful high-resolution tools for studying the underlying pathologies and neural networks associated with different forms of epileptogenesis (Buzsáki and Silva, 2012; Ibrahim et al., 2014; Williams et al., 2016).

The epileptogenicity of FCD is likely related to an imbalance in the level of excitation to inhibition in the affected brain regions (Redecker et al., 1998; Avoli et al., 1999; Zhu and Roper, 2000; Eichler and Meier, 2008) The hyper-excitable zone of brain tissue can extend several millimeters from the core microgyric lesion (Jacobs et al., 1996, 1999b, Jacobs et al., 2009, 2012; Luhmann and Raabe, 1996; Roper et al., 1997; Redecker et al., 1998) and has been associated with alterations in expression of excitatory and inhibitory receptors in dysplastic and exfocal regions of freeze lesioned cortex (Zilles et al., 1998; Redecker et al., 2000). In particular, hyperexcitable regions are isolated to regions outside the microgyric core as experimental isolation of ectopic tissue or microgyria fail to remove the hyperexcitability (Jacobs et al., 1999a). In fact, accurate identification and complete resection of hyper-excitable tissues surrounding the microgyric lesion is a key factor that determines seizure-free outcome in FCD patients (Fujiwara et al., 2012; Guerrini and Dobyns, 2014; Moosa and Gupta, 2014). To date, the cellular and circuit features underlying paroxysmal discharges *in vivo* still have not been elucidated.

The aim of the current study was to assess the altered *in vivo* cortical neurophysiology of a recently characterized FCD model shown to be associated with the high prevalence of spike-wave discharges (SWDs; Sun et al., 2016). In this model, a distinct cortical microgyric cleft is consistently observed that results in close to a 90% incidence of SWDs in adult animals (Sun et al., 2016) similar to the neuropathology observed in FCD patients exhibiting cortical microgyria that also exhibit a high incidence of epilepsy (Luhmann, 2016). Here, we provide a comprehensive spectrotemporal analysis of the malformed cortex following hyper-excitable activation using anesthesia-induced burst-suppression (B-S; Williams et al., 2016). In our initial study (Williams et al., 2016) we found that this transitional state of anesthesia-induced hyper-excitability is significantly enhanced in animals exposed to a neonatal freeze lesion and often contains spike-wave components similar to that observed during SWDs in awake animals. In the current study, we extend our initial findings and focus on the incidence and laminar distribution of HFOs, one- and two-dimensional spectrotemporal mapping of altered local field potentials (LFPs), and characterization of hyperexcitable single-unit distributions across cortical lamina using commercially available linear micro-electrode arrays. Research into the underlying circuitries that control hypersynchronous activity as well as possible differential patterns between epileptic conditions that arise in disparate parts of the brain will be critical for understanding and ultimately treating these activities.

## Materials and Methods

All experiments were performed under protocols approved by the Institutional Animal Care and Use Committee (IACUC) of the University of Wyoming. Animals were housed in a vivarium maintained at 22–23°C on a 12:12 h light-dark cycle. Food and water were available *ad libitum*.

### Freeze Lesion Model

**Figure 1:**
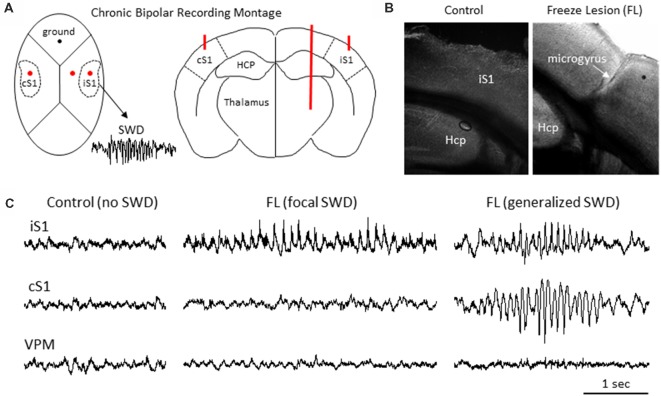
Freeze lesion induced microgyrus and local field potential (LFP) recordings of spike-wave discharges (SWDs). **(A)** Diagram of mouse skull (left) and coronal brain section (right) indicating position of the chronic bipolar recording electrodes in the ipsilateral S1 (iS1), contralateral S1 (cS1) and thalamus. HCP, hippocampus. **(B)** Representative coronal section from the S1 cortical region of a control and FL mouse brain. **(C)** Examples of focal and generalized SWDs from FL animals that were generally confined to cortical regions and not observed in control animals.

Neonatal freeze lesions were induced in P0–1 pups (male and female CD-1) using a modification of the model described by Dvorak et al. (1978) to induce neocortical microgyria (right S1 somatosensory region) as previously described (Sun et al., 2016). Briefly, mice pups were immersed in wet ice until movement and response to tail pinch was absent. The skull was then exposed through a midline scalp incision and a freezing probe with a 1 mm diameter circular tip (cooled to −196°C in liquid nitrogen) was placed on the skull over the somatosensory cortex, 2 mm lateral to midline and 0.5 mm caudal to the Bregma for 1–2 s. This procedure was routinely completed within 5 min and resulted in the development of a consistent microgyrus in the adult brain (e.g., Figure 1A). Control animals underwent a sham surgery (exposed to all surgical procedures minus the freeze lesion).

### Extracellular Microelectrode Array Studies (Anesthetized Mice)

Extracellular signals were recorded using 16-electrode linear micro-electrode SmartProbe^TM^ arrays (NeuroNexus, Ann Arbor, MI, USA) from the right S1cortical region of anesthetized control (sham) and freeze lesion mice as previously described (Williams et al., 2016). The electrode array consisted of a single shank with 16 individual iridium electrodes (A1 × 16, 3 mm length, 15 μm thickness, each electrode separated by 100 μm space) with on-board electronics for digital conversion of the signals and linked to a SmartBox^TM^ control and data streaming system through a SmartLink headstage (NeuroNexus). These electrodes typically have a impedance of around 1.5 MΩ. The array was positioned perpendicular to the cortical surface to allow recording from both cortical and subcortical brain structures. Each signal was digitally filtered (1–10,000 Hz band-pass and 60 Hz notch filters), and recorded at a sample rate of 30 kHz using Smartbox 2.0 software (NeuroNexus). Additional off-line digital filtering was used to define LFPs (1–100 Hz) and multi-unit activity (MUA; 300–3,000 Hz). All recordings were obtained during B-S due to the hyperexcitable state of the brain during this level of anesthesia (Kroeger and Amzica, 2007; Amzica, 2009; Ferron et al., 2009), recently characterized in the freeze lesion model to be sensitive to changes in hyperexcitability of the epileptic brain (Williams et al., 2016). In brief, mice were anesthetized under isoflurane anesthesia (2%, delivered in oxygen) and secured in a stereotaxic frame (Stoelting, Wood Dale, IL, USA). A scalp incision was made to expose the skull over the right S1 area. The micro-electrode array was then advanced at an angle of 40° from vertical (~1650-micron depth) into the S1 cortex (see Figure 1A) using a hydraulic micromanipulator (Narishige, Amityville, NY, USA). A reference electrode (silver wire) was placed in the skin flap of the scalp incision. The isoflurane anesthesia level was then reduced to 1.0% to induce B-S. Extracellular recordings began once stable baseline activity was observed. Signals were digitally filtered (1–7,500 Hz band-pass and 60 Hz notch filters), and recorded at a sample rate of 20 kHz using Smartbox 2.0 software (NeuroNexus).

### Bipolar Field Potential Recordings (Unanesthetized Mice)

A screw free, glue-based electrode assembly system (Wu et al., 2008) was used to record LFPs from the brains of awake, behaving animals as previously described (Sun et al., 2016). In brief, mice were anesthetized (2% isoflurane, delivered in oxygen) and secured in a stereotaxic frame (Stoelting, Wood Dale, IL, USA) for electrode implantation. Bipolar electrodes were constructed of twisted pairs of polyimide-insulated stainless steel wires (125 μm diameter, California Fine Wire Co., Grover Beach, CA, USA) and connecting pins (Digikey, Thief River Falls, MN, USA). The electrodes were stereotaxically implanted through a small burr hole in the skull over the ipsilateral (right) S1 cortex (1.5 mm posterior and 3.5 mm lateral to Bregma) and glued in place with dental cement. A ground electrode was placed into the ipsilateral olfactory bulb area. Recording sessions were performed over 6–24 h with automated infrared activity tracking to detect animal movement in a circular recording arena that allowed for free movement of the animal. Electrographic signals were amplified (Model 1700 differential AC amplifier, A-M system, Carlsborg, WA, USA), digitized (Power 1401 A-D converter, Cambridge Electronic Design Limited, Cambridge, England), and continuously recorded (Spike2 software, Cambridge Electronic Design Limited, Cambridge, England) at a sample rate of 3.2 kHz.

### Histopathology

Following completion of electrophysiology recordings, animals were anesthetized and transcardially perfused with 0.9% saline followed by 4% paraformaldehyde for histological analysis as previously described (Williams et al., 2016). In brief, brains were extracted, immersed in 4% paraformaldehyde for 24 h, and then transferred to 0.1 M phosphate buffer containing sequentially increasing levels of sucrose (10/20/30%, pH 7.4, 4°C) across three consecutive days. Brain issue was then cut into serial sections through the area of interest (50–300 μm thick). The back of the micro-electrode was swabbed with DiI (2 mg/ml in ethanol, Invitrogen Molecular Probes, Eugene, OR, USA) for visualization of the electrode track (e.g., Figure 1A). All sections were then mounted on glass slides with a DAPI mounting medium (Vectashield, Vector Laboratories, Burlingame, CA, USA), evaluated under a light/fluorescent microscope (Zeiss Axioskop 2, Ontario, CA, USA), and digitally imaged using Axiovision software (ver. 4.6, Zeiss).

### Stereotaxic Viral Injection and Optogenetic Stimulation *in vivo*

Virus injection was performed in sham or FL mice aged P14-P16. Mice were anesthetized with 2% isoflurane (vol/vol) and maintained with oxygenated 1% isoflurane throughout surgery procedure. AAV2.1.CAG.hChR2(H134R)-mCherry (University of Pennsylvania Vector Core) was used. Viral vector (2 μl) was loaded into the tip (~10 μm in diameter) of a beveled glass micropipette (Drummond Scientific Co., Broomall, PA, USA). A custom stereotactic apparatus was used to deliver viral vector to cortex through a small hole drilled into the skull. For local vS1 injection, virus was injected at two depths: 400 μm and 800 μm; for L4 injection, 400 μm and 600 μm. For each depth, a volume of ~150 nl was injected within 1–2 min using a micromanipulator (MP-285-system, Sutter Instrument). Injection pipette was kept in place for 5 min for each depth after injection. Injected mice were put back to dam after 5–10 min recovery from anesthesia in a separate cage with a heating pad underneath. After weaning day, pups were separated and housed by gender until experiments. *Optogenetic*. We placed a multi-mode fiber optic patch cable *via* a 1.25 mm OD multimode ceramic zirconia ferrule (Precision Fiber Products, Inc., Milpitas, CA, USA) near the recording site. The multi-mode fiber optic patch cable was coupled to a blue laser which is triggered by a custom written program.

### Data and Statistical Analysis

**Figure 2:**
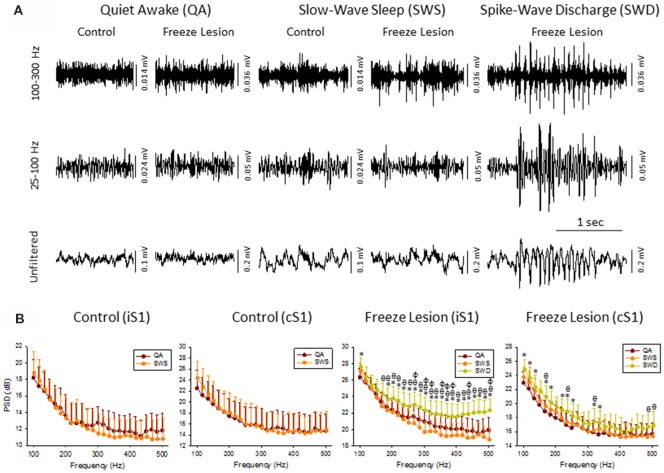
High frequency oscillations (HFOs) across vigilance state and during SWD. **(A)** Sample LFP recordings of quiet awake (QA), slow-wave sleep (SWS), and SWDs in control and FL animals (iS1 recordings). Digital filtering of the field potential recordings revealed that SWDs were associated with an increased in HFOs (upper traces) as compared to the full spectrum recordings (lower traces). **(B)** Comparison of spectral power (i.e., PSD) levels across vigilance state and during SWDs in control (*n* = 5) and FL (*n* = 5–10) animals. *P < 0.05 SWD vs. QA, ^θ^*P* < 0.05 SWD vs. SWS, ^φ^*P* < 0.05 QA vs. SWS (Holm-Sidak *post hoc* analysis).

All recorded electrographic signal files were exported to NeuroExplorer (Nex Technologies, Madison, AL, USA) for off-line data analysis and visual inspection by an experimenter blinded to the test group. Each signal was digitally filtered using finite impulse response filters to define changes across a continuous array of frequency bands dependent on the sampling rate of the signals; low frequency (<25 Hz), gamma (25–100 Hz), low-frequency ripple (LFR; 100–300 Hz), high-frequency ripple (HFR; 300–800 Hz), and MUA (800–5,000 Hz). HFOs were identified as amplitude increases in the digitally filtered low and HFR bands (as demonstrated in Figure 1B) followed by a spectrograph analysis to evaluate increases in high-frequency spectral power (as demonstrated in Figure 2), a protocol similar to the techniques used for clinical detection of HFOs in epileptic patients (Zijlmans et al., 2012). Quantitative spectral analysis of discrete extracellular artifact-free recording samples (2–5 s duration) were analyzed during periods of B-S or SWDs to evaluate spectrotemporal changes in PSD and LFP coherence between channels using a previously described protocol (Williams et al., 2016).

Single-unit spike sorting of linear array recordings was conducted off-line with Spike2 using a combined template matching and principal component analysis protocol. Spike waveforms greater than 2 standard deviations above threshold were initially separated using automated template matching. Manual verification of separated spike waveforms was used to ensure consistent waveforms with refractory periods greater than 1 ms between spikes. Following initial separation of spikes, principal component analysis was used to classify neurons into well isolated groups with clearly separated cluster boundaries in 3-D space across each channel. Firing phase analysis was used to compare the firing rate of isolated single-units to distinct frequency oscillations: (1) spike field coherence (SFC) analysis was used to compare single-unit firing rate to specific frequencies across the LFP derived from the same signal; and (2) zero-phase oscillation timestamps were computed for each frequency band of interest and compared to individual single-unit spike rates to construct spike-phase histograms.

Following primary analysis, raw data was exported to Microsoft Excel for tabulation of statistical averages and standard error values or Sigmaplot (SYSTAT software Inc., San Jose, CA, USA) for graphical display (including spectral heat maps) and statistical analysis between groups. A multifactorial ANOVA was used to evaluate main effects and interactions when multiple independent variables were present followed by a Holm-Sidak *post hoc* analysis to evaluate significant differences between individual groups. A Fisher’s Exact Test was used to assess significant differences in the relative proportion of responses between experimental groups. A *P* value of < 0.05 was considered significant.

## Results

Intracranial bipolar electrodes or linear micro-electrode arrays were used to evaluate electrophysiological disruption of the FL brain, with a particular focus on changes in the ultra-high frequency range (>100 Hz), in a total of 45 mice of either sex on a CD-1 background (12 controls: six male and six female, weight 33 ± 4 g; and 33 FL: 20 female and 13 male, weight 35 ± 4 g) ranging in age from 5 to 11 months old (8 ± 1 months). More detailed animal information for each experiment is provided in relevant “Results” section. Upon completion of the electrophysiological recordings, brain tissue was collected from a subset of animals to verify that animals exposed to a neonatal FL (*n* = 7) exhibited a distinct microgyrus within the right S1 region of the brain as compared to the normal cortical morphology of control animals (*n* = 6; Figure 1B).

### Chronic Bipolar Field Potential Recordings (Unanesthetized, Fully Behaving Mice)

LFP recordings from three brain regions [ipsilateral S1 (iS1), contralateral S1 (cS1), and VPM, see Figure 1A] were evaluated for the presence of SWDs. Average age was 6.40 ± 0.98 months for control animals (*N* = 5 mice, weight = 35 ± 3 g, three male and two female) and 8.67 ± 0.54 months for FL animals (*N* = 17 mice, *P* > 0.05 between groups for age, 11 male and six female, weight = 33 ± 2 g). SWDs were defined as 10–12 Hz repetitive SWDs above baseline activity of at least 1 s in duration that occurred primarily during slow-wave sleep (SWS) as previously described in detail for this mouse model (Sun et al., 2016). Control animals exhibited no evidence of SWDs (0/5) while 83% (10/12) of FL animals exhibited either focal (5/10 recordings) or generalized (5/10 recordings) SWDs over the course of the recording session of about 12 h in each animal (Figure 1C). SWDs were confined to the cortical regions and were not evident in the subcortical (i.e., VPM) recordings in any of the 10 animals exhibiting SWDs.

Cortical LFP recordings were band-pass filtered to evaluate changes in HFOs across vigilance state and during SWDs (Figure 2A). Control animals exhibited relatively stable levels of background activity across periods of quiet awake (QA) and SWS (Figure 2A) with no significant differences in spectral power (100–500 Hz) in either brain hemisphere (Figure 2B). Freeze lesion animals exhibited similar levels of activity during QA and SWS, as compared to controls (Figure 2A) though there was a significant increase in spectral power between the two vigilance states above 250 Hz in the ipsilateral but not contralateral hemisphere (Figure 2B). In contrast to QA and SWS, a transient/oscillatory increase in HFO amplitude was observed during periods of SWD (Figure 2A). Significant increases in spectral power were also measured between SWDs and either vigilance state in both cortical hemispheres though stronger increases in PSD were observed in the iS1 as compared to cS1 (Figure 2B), particularly for frequencies above 200 Hz that exhibited a 2–3 dB increase in spectral power as compared to either QA or SWS.

The increase in HF activity (>100 Hz) that occurred during SWDs presented as oscillatory wax-wane amplitude alterations locked to the spike-and-wave pattern of the SWD (Figure 2A).

Both SWDs and hyperexcitable B-S both show increases in HFR activity that were phase-locked to the high-frequency spike pattern of hyperexcitable brain waves occurring at the trough of low-frequency waves.

**Figure 3:**
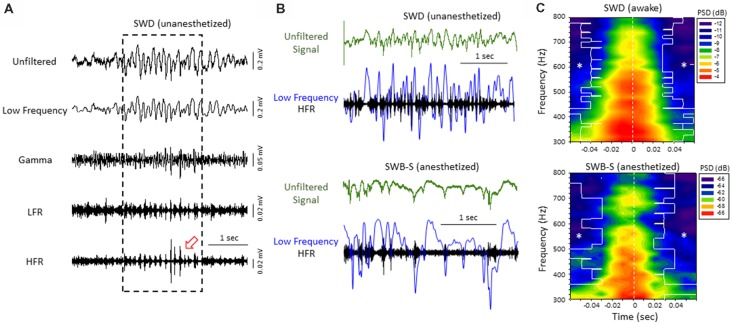
High-frequency ripple (HFR) activity recorded from the iS1 cortex was locked to high-amplitude spike deflections observed in the low frequency band of both awake and anesthetized animals. **(A)** Example of a spontaneous SWD (awake) from the iS1 cortex and corresponding HFOs. Each signal was digitally filtered into spectral bandwidths for comparison as defined in Figure 1. Strong wax-and-wane amplitude oscillations (red arrow head) were commonly observed across the HFR spectrum locked to the large amplitude spike deflections of the SWD. **(B)** Representative traces from freeze lesion animals indicating that the oscillatory amplitude changes observed in the HFR band were locked to the spike-and-wave pattern of the low frequency band during both spontaneous SWDs (upper panel) and spike-wave burst-suppression (SWB-S) events (lower panel). In each example an overlay of the low frequency (blue trace) and HFR (black trace) bands are presented below each unfiltered trace (green) for comparison. **(C)** Average perievent spectrograms indicating that the increases in PSD within the HFR frequency band were centered on the “spike” of the spike-and-wave pattern that occurred during either SWDs (upper panel, *n* = 5) or B-S events (lower panel, *n* = 6). SWDs were recorded from the iS1 region of awake animals while the B-S events shown were recorded from the supragranular layer of the iS1 region of anesthetized animals. *P < 0.05 compared to PSD values at time 0 for each frequency bin (Holm-Sidak *post hoc* analysis).

LFP recordings from the iS1 cortex of 11 *unanesthetized* animals (six males and seven females, age = 8 ± 2 months, weight = 31 ± 2 g) exposed to neonatal freeze lesion injury, and seven sham-treated controls (three male and four female mice, age = 7 ± 1 months, weight = 32 ± 2 g) were evaluated for the presence of SWDs and associated HFOs for comparison to the results obtained in the B-S model (i.e., anesthetized animals). SWDs were defined as 10–12 Hz repetitive SWD patterns above baseline activity of at least 1 s in duration that occurred primarily during SWS as previously described in detail for this mouse model (Sun et al., 2016). The cortical field potential recordings were band-pass filtered to evaluate changes in HFOs across frequency bandwidths up to 800 Hz (Figure 3A). In comparison to periods of non-ictal activity, a distinct pattern of HFO activity was observed in all freeze lesion animals during periods of SWD that presented as stereotypical transient/oscillatory increases in signal amplitude, particularly within the HFR band, locked to the SWD pattern (Figure 3A).

Direct comparison of the two forms of hyperexcitable activity observed from the same mouse FCD model indicated that amplitude-modulated increases in HFR activity occurred during either SWDs in unanesthetized, behaving animals or hyperexcitable spike-and-wave patterns observed during B-S in anesthetized animals. In both forms of epileptiform activities, the high frequency oscillatory amplitude modulations observed in the HFR band were phase locked to large amplitude spike deflections of the low frequency band (LFR) as demonstrated in Figure 3B. Average perievent histograms of changes in PSD (300–800 Hz) verified that the increases in HFR spectral power were centered on the spike deflections during either SWDs or B-S (Figure 3C). These data shows that the frequency distribution pattern of hyperexcitable B-S activities in anesthetized animals are similar to that of SWD in unanesthetized, behaving animals, and thus indicate a similar underlying neural mechanisms that were revealed in earlier results.

### Neonatal Freeze Lesions Induced Laminar-Specific Increases in HFOs in the Malformed Cortex

**Figure 4:**
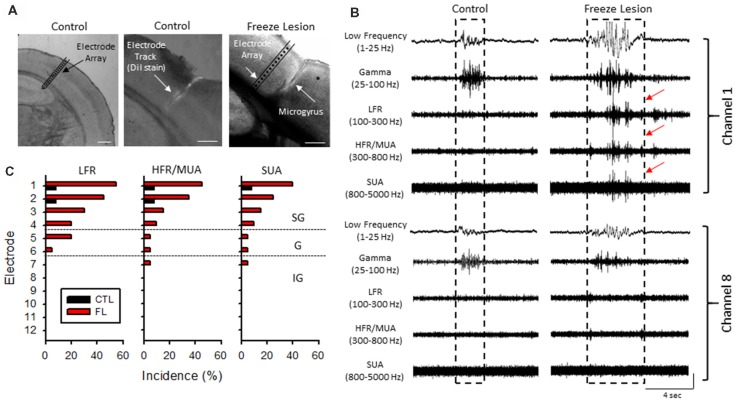
Band-pass filter analysis of linear array recordings indicated an increase in HFOs during periods of B-S in freeze lesion animals in the upper cortical layers of the malformed cortex. **(A)** Representative coronal sections from the S1 cortical region of control and freeze lesion animals. Left panel indicates orientation of the linear micro-electrode array in comparison to the S1 cortex. The first 12 electrodes spanned the vertical extent of the cortex. Middle panel indicates the micro-electrode recording track as indicated by fluorescent DiI labeling of the linear array. Right panel demonstrates a freeze lesion induced microgyrus as compared to the position of a micro-electrode array. White scale bars = 400 μm. **(B)** Representative samples of B-S events recorded from channels 1 and 8 of a control and freeze lesion animal. The waveforms were digitally filtered to evaluate spectral changes across defined bandwidth ranges; low-frequency, gamma, low-frequency ripple (LFR), HFR, and multi-unit activity (MUA). Increases in HFOs above 100 Hz were largely confined to the upper cortical layers near the site of the freeze lesion injury (red arrows). Amplitude scale bar = 0.4 mV (unfiltered signals) and 0.04 mV (filtered signals). Amplitude of filtered signals was increased to show detail of high-frequency events. **(C)** Percent of recordings exhibiting increases in HFOs between control and freeze lesion animals across three frequency bands. Electrodes 1–12 represent recordings from the upper to lower regions of the cortex, respectively. SG, supragranular; G, granular; IG, infragranular.

**Figure 5:**
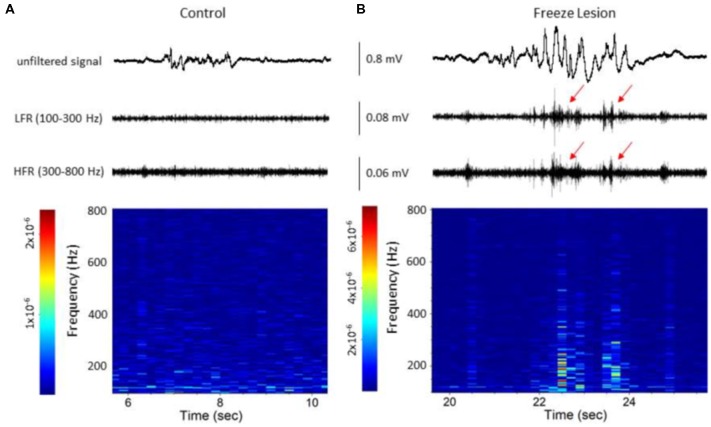
Spectrograph analysis of B-S events demonstrating the increase in high-frequency spectral power that was observed during the HFOs. **(A)** B-S event from a control animal indicating a lack of HFOs (i.e., LFR and HFR, upper panel) and minimal change in high-frequency spectral power (spectrograph, lower panel). **(B)** B-S event from a freeze lesion animal indicating the presence of HFOs (red arrows, upper panel) and subsequent increase in high-frequency spectral power (spectrograph, lower panel). **(A,B)** The corresponding signal is presented above each spectrograph. Recordings are representative samples from the most superficial linear array electrode (CH01) of both a control and freeze lesion animal. Scale bar represents the PSD (dB) level for each spectrotemporal subunit.

Acute cortical field potential recordings were obtained using linear microelectrode arrays during hyperexcitable epochs of B-S in anesthetized mice (Williams et al., 2016) from one or more distinct electrode penetrations within the iS1 cortex of seven control and 11 freeze lesion animals. Average age was 7.93 ± 0.87 months for control animals and 8.41 ± 0.84 months for freeze lesion animals (*P* > 0.05 between groups). The location and orientation of the microelectrode array in relation to the S1 region is demonstrated in Figure 4A (left panel). DiI labeling was use to verify the location of the electrode track as demonstrated in Figure 4A (middle panel). A distinct microgyrus was observed in animals exposed to the neonatal freeze lesion injury (7/7 animals) as compared to the intact tissue of control animals (0/7 animals; Figure 4A, right panel). Using this technique, the electrode array was positioned in the iS1 cortex, within 0.5–3 mm of the microgyrus as verified from histological analysis of the tissue (Williams et al., 2016). Digital filtering of raw signals across defined frequency bands was used to evaluate presence of HFOs during B-S (Figure 4B). Visual inspection of B-S activity indicated that, in comparison to sham controls, freeze lesion animals exhibited an increase HFO activity above 100 Hz (up to 5,000 Hz) as indicated by transient increases in signal amplitude above baseline that was largely confined to the upper cortical layers (Figure 4B, channel 1 vs. 8). In total, only 9% (1/12) of controls exhibited increases in HFOs above 100 Hz as compared to 60% (12/20) of freeze lesion recordings during periods of B-S (*P* < 0.05 between groups, Fisher’s Exact test) with a similar profile of activity across cortical level for the HFO frequency bands above 100 Hz as summarized in Figure 4C. Spectrograph analysis of each B-S event was also used to verify that the presence of HFOs was associated with subsequent increases in high-frequency spectral power as demonstrated in Figure 5. In all cases, the presence of HFOs and subsequent increases in high-frequency spectral power were only observed during periods of hyperexcitable burst activity and were not observed during the intermittent periods of signal suppression.

### Spectral Disruption of Cortical S1 Circuitry in Freeze Lesion Brain

**Figure 6:**
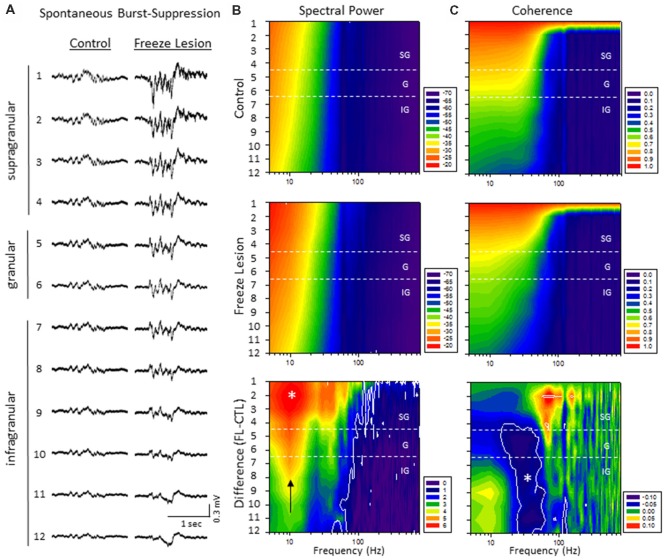
A significant increase in spectral power and altered spectral coherence pattern were observed in the malformed cortex of freeze lesion animals as indicated from spectral analysis of linear electrode array recordings of anesthesia induced B-S. **(A)** Representative micro-electrode recordings of B-S events across cortical lamina from a control and freeze lesion animal under general anesthesia (2% isoflurane). **(B)** Average spectral power maps of control, freeze lesion, and the difference between each map. Black arrow indicates peak 10 Hz increase in PSD values from freeze lesion vs. control animals. **(C)** Average spectral LFP coherence maps of control, freeze lesion, and the difference between each map. **(B,C)** Values represent the group means from control (*n* = 12) and freeze lesion (*n* = 20) recordings. The topographic lines (solid white) indicate the *P* < 0.05 cutoff threshold and the white asterisks designate regions significantly different between groups (Holm-Sidak *post hoc* analysis). See “Results” section for additional statistical analysis.

Quantitative analysis of changes in the spectral architecture of the malformed cortex was evaluated by spectral analysis of control and freeze lesion animals during periods of B-S (Figure 6). Overall, B-S events typically presented across all layers of the S1 cortex in both control and freeze lesion animals (Figure 6A). Significant differences in average PSD values were measured across both frequency and electrode for both control and freeze lesion animals (*P* < 0.05, multivariate ANOVA, Figure 6B). In general, both experimental groups exhibited a similar expression pattern of PSD values with strongest increases in the upper cortical layers and the majority of spectral power confined to frequencies below 30 Hz (Figure 6B, upper two panels). However, comparison across groups (i.e., control vs. freeze lesion) indicated that freeze lesion animals exhibited significant increases in PSD of up to 6 dB predominately below 80 Hz but extending up to 800 Hz, particularly in the upper cortical layers (*P* < 0.05, Holm-Sidak *post hoc* analysis, Figure 6B, lower panel). In freeze lesioned animals, peak PSD increases were observed in the alpha band (8–12 Hz; Figure 6B, black arrow, lower panel), a peak frequency range increase also observed during SWDs in this model thought to be associated with seizure generalization and synchronization across brain regions and hemispheres (Sun et al., 2016).

The LFP coherence of each recorded signal (referenced to channel 1) was used to evaluate the similarity in waveform patterns across cortical lamina. Channel 1 was chosen as the reference channel as is represents the upper most cortical layer that would dominate cortical EEG recordings and also indicated the most robust B-S activity in both control and freeze lesion animals (i.e., Figure 6A). Results indicated significant differences in LFP coherence across both frequency and electrode position for both control and freeze lesion animals (*P* < 0.05, multivariate ANOVA, Figure 6C). Both experimental groups exhibited a similar pattern of LFP coherence with a gradual drop in coherence values as the distance between the electrodes increased (i.e., lowest values for the deepest cortical layers) along with a sharp drop in coherence values above 50 Hz (Figure 6C, upper two panels). However, comparison of LFP coherence across groups (i.e., control vs. freeze lesion) indicated a differential change dependent on frequency and electrode depth. In comparison to controls, freeze lesion animals exhibited a significant increase in LFP coherence in the supragranular layer for signals above 50 Hz (50–200 Hz) and contrasting drop in LFP coherence in the lower granular and infragranular layers for signals lower than 70 Hz (15–70 Hz; *P* < 0.05, Holm-Sidak *post hoc* analysis, Figure 6C lower panel) suggesting an altered communication network between cortical layers of the malformed cortex (see “Discussion” section).

### Laminar Profile of Spontaneous Single-Unit Activity Induced by Neonatal Freeze Lesions

**Figure 7:**
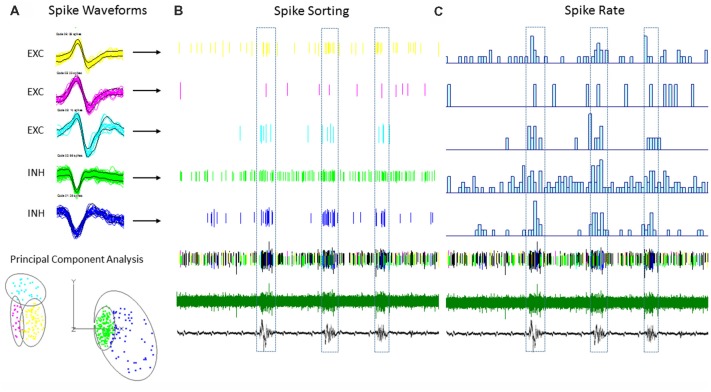
An example of single-unit activities (SUAs) sorting during the B-S activities in a FL mouse.** (A)** Five (SUAs, three EXCs and two INHs) were shown from a single linear electrode recording. The spike amplitude and duration of these SUAs formed two separate large clusters using principal component analysis, INHs and EXCs are clustered together. **(B)** Raster plot showing the spike time of each spike aligned to the B-S LFPs. **(C)** The spike rate plotted on top of the LFPs. The spike rates are higher for all SUAs during the bursts activities.

**Figure 8:**
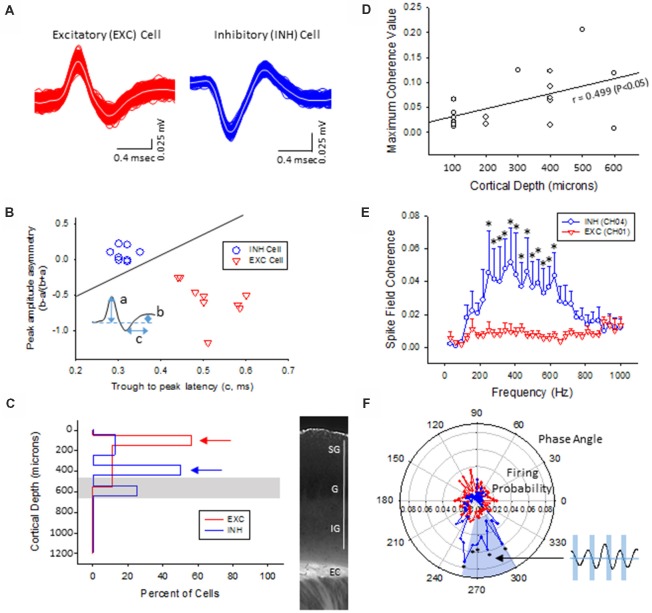
Inhibitory cells from the lower supragranular layer were phase-locked to HFR oscillations in the malformed cortex of freeze lesion animals. **(A)** Spike sorting of a putative excitatory (red) and inhibitory (blue) cell, based on the associated waveform of each spike. White line in each trace represents the average of the multiple overlapping traces shown in color. **(B)** Three parameters were calculated from the average trace of each spike waveform (a–c as indicated from inset) to extract features used to classify neurons as either excitatory or inhibitory. Solid line represents the cutoff used to classify excitatory vs. inhibitory cells (Reyes-Puerta et al., 2016). **(C)** Distribution of isolated single-units across cortical depth. Value of 0 = cortical surface. Arrows indicate the cortical level that expressed the maximal number of excitatory vs. inhibitory neurons. Gray bar indicates level of the granular layer. Representative section of the S1 cortex is shown in the right panel for comparison. White bar = 1,000 μm. EC, external capsule. **(D)** Pearson’s correlation between maximum spike field coherence (SFC) values and cortical depth. **(E)** Average SFC values between excitatory (CH01, *n* = 5) and inhibitory cells (CH04, *n* = 4) across frequency bin. *P < 0.05, Holm-Sidak *post hoc* analysis between groups. **(F)** Polar plot of average firing probability of excitatory (CH01, *n* = 5, red) and inhibitory (CH04, *n* = 4, blue) units across phase angle of the HFR spectrum (300–800 Hz). Inhibitory cells indicated phase-locked responses (blue shaded region) to the downward phase of the corresponding ripple wave (i.e., see inset). *P < 0.05, Holm-Sidak *post hoc* analysis between groups.

Multi-units like spike activities (MUA, >300 Hz) were recorded from multiple sites in 7/7 sham treated animals (three males and four females, age = 7 ± 2 months, weight = 34 ± 2 g) and 12/12 FL animals (six males and six females, age = 8 ± 2 months, weight = 35 ± 2 g) during the spontaneous B-S period. The MUA in each animal were numerous and were not able to be separated for further analysis (e.g., Figure 5). Therefore, well isolated single-units (Sirota et al., 2008; Reyes-Puerta et al., 2016) were used to classify and map the location of spontaneously firing cells that occurred during periods of B-S across cortical lamina. Overall, detection of spontaneous unit activity was rare in the S1 region of control as compared to freeze lesion animals and no single-units were isolated from any of the control recordings (11 recordings, seven animals) while 17 well isolated single-units were recorded from the S1 region of freeze lesion animals (20 recordings, 12 animals). As such, all subsequent analysis was based on single-units isolated from freeze lesion animals. Single-units were classified as either putative excitatory (*n* = 9) or putative inhibitory (*n* = 8) cells based on their waveform pattern (Figure 7) using a previously published classification protocol (Reyes-Puerta et al., 2016). The isolated excitatory (EXC) and inhibitory (INH) single-units were validated with principal component analysis and shows (Figure 8A) complete separation as expected in both the same recordings from the same animals (e.g., Figure 7) or all animals (Figure 8B). Although all isolated single-units were confined to the upper cortical layers of the malformed cortices (electrodes 1–6), a differential distribution of excitatory vs. inhibitory cells was observed across cortical lamina (Figure 8C). The majority of excitatory cells (78%, 7/9 cells) were recorded from the upper cortical layers (channels 1–3) while the majority of inhibitory cells (75%, 6/8 cells) were recorded from deeper cortical structures including the granular layer (channels 4–6).

### Phase-Locking of Putative Inhibitory Cells to HFOs

**Figure 9:**
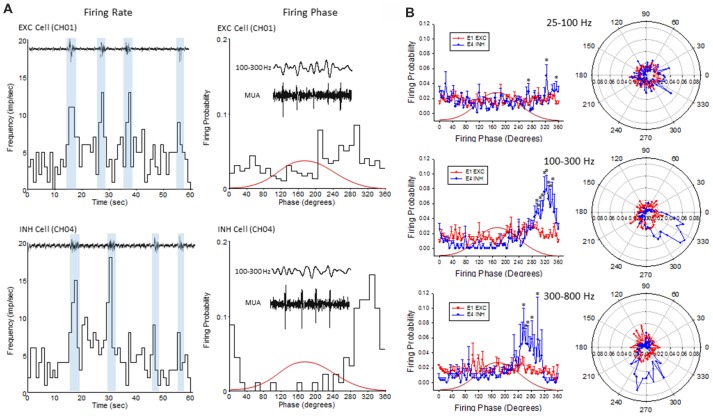
Firing phase analysis between EXC units recorded from channel 1 (CH01) and INH units recorded from (CH04) across HFO bandwidths. **(A)** Firing rate (left column) of exemplar EXC (top) and INH (bottom) single-units in comparison to B-S activity. Blue bars indicate the increase in firing rate during B-S events. Firing phase (right column) of these same units in relation to HFOs (100–300 Hz). **(B)** Average firing phase (left column) of EXC (CH01, *n* = 5) and INH (CH04, *n* = 4) units across three frequency distributions. *P < 0.05 between groups (Holm-Sidak *post hoc* analysis). The same data is presented in polar plots (right column) to demonstrate the relative phase-locking of average unit activity across each frequency spectrum. Red curves in **(A,B)** indicate the relative phase of the associated waveform.

The plot of spike rates of individual units in most animals indicated an enhanced spiking during the LFP bursts (Figures 7C, 9A). Next, SFC analysis was used to assess if single-unit firing patterns correlated to specific frequencies within the LFP including HFOs up to 1 kHz. Single-unit SFC values were averaged across electrodes 1–2, 3–4, and 5–6 to evaluate laminar differences. Significant increases in SFC values were observed in cells from the deeper cortical regions as compared to the most superficial electrodes; 188–468 Hz (electrodes 3–4) and 344–531 Hz (electrodes 5–6) as compared electrodes 1–2 (Holm-Sidak *post hoc* analysis). A Pearson’s correlation analysis also indicated a significant increase in maximum SFC values across recording electrode depth (Figure 8D). Given the differential laminar distribution across cell type, a subset analysis was used to compare SFC values between the upper excitatory cells recorded from channel 1 (*n* = 5) and the lower inhibitory cells recorded from channel 4 (*n* = 4) that represent the cortical location of the highest percentage of cells recorded for each cell type (i.e., Figure 8C). Results indicated only weak SFC for the excitatory cells located in the superficial cortical layers as compared to the significantly higher values measured from the deeper inhibitory cells, specifically for frequencies above 250 Hz (Figure 8E).

To further address the correlation between unit firing and HFOs a firing phase analysis was used to evaluate possible phase-locking of unit activity across different frequency bandwidths. Both EXC (CH01) and INH (CH04) cells exhibited increases in firing rate during periods of B-S as demonstrated in Figure 9A (left column), however, only INH (CH04) cells indicated strong phase locking to HFO phase as demonstrated in Figure 9A (right column). Statistical analysis across groups indicated significant increases in firing probability in the higher HFO ranges (100–300 and 300–800 Hz, *P* < 0.05) but not for the lower gamma range (25–100 Hz, *P* > 0.05; multivariate ANOVA) although the results were dependent on cell type (*P* < 0.05, interaction between frequency × phase). *Post hoc* analysis indicated that the INH (CH04) cells had significantly higher firing probabilities across the 100–300 and 300–800 Hz oscillations, confined to the downward slope of the individual waveform (i.e., phase angles ranging between 250 and 350°, Figure 9B, left column). Polar plots are also presented to demonstrate the strong phase locking of the INH cells across the high-frequency range (>100 Hz) associated with ripple activity (Figures 8F, 9B, right column).

**Figure 10:**
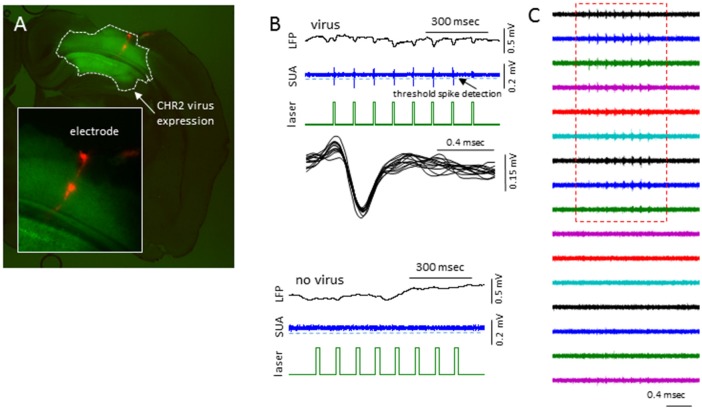
Laser activation induces reliable SUAs in the sham-treated animals.** (A)** Photomicrograph of brain slice showing the channel-rhodopsin 2 (ChR2) transfected S1 region and location of linear electrodes across the center of the ChR2 transfected area. **(B)** In AAV-ChR2 transfected sham-treated animals, repeated exposure to short blue laser pulses (0.2 s), reliably and repeatedly induced SUAs in the S1 of ChR2 transfected brain but not in the slices without ChR2 expression. **(C)** The laser evoked SUA were distributed in the top 9/12 cortical sites.

In sham treated animals, well-isolated single-units were rare (2/7 mice), presumably because the suppression of activities associated with the anesthesia. To further validate if this was the case, we expressed channel-rhodopsin 2 (ChR2) in S1 of shame treated mice and examined the evoked single-units activities (SUAs; Figure 10). In 5/5 mice examined, blue laser (480 nm) delivered locally *via* a fiber optic positioned next to the recording site reliably evoked SUAs in 5/5 mice in majority of the electrode sites in the cortex (Figure 10) under all anesthesia levels (0.5%–2% isoflurane) thus the lack of the SUAs in sham-treated animals were unlikely to be caused by damage or some artifact.

## Discussion

In the current study, we evaluate the response of the malformed cortex to hyper-excitable activation due to anesthesia-induced B-S. Utilization of the B-S model represents a novel method to explore neural hyperexcitability in epileptic brain and has recently been characterized in the neonatal freeze lesion model (Williams et al., 2016). B-S is a transitional brain state of induced hyper-excitability that occurs between signal suppression (deep anesthesia) and slow-wave activity (light anesthesia) that can be induced in both humans and animals by a variety of anesthetics with different modes of action (Kroeger and Amzica, 2007; Ferron et al., 2009). Previous studies have indicated that the incidence and spectral power of B-S events are significantly increased in freeze lesion animals, confined to the upper cortical layers, and often contain spike-and-wave components similar to the SWDs observed in unanesthetized animals (Williams et al., 2016). In the current study, we show that the upper cortical region of the malformed cortex is also associated with a significant increase in HFOs similar to that observed during SWDs in unanesthetized animals and are phase-locked to laminar-specific inhibitory cells within the deep supragranular layer.

The use of high-density linear micro-electrode arrays allowed for cross-laminar mapping of the S1 cortex and indicated distinct disruptions in spectrotemporal architecture near the site of the freeze lesion-induced microgyrus. In particular, a significant increase in spectral power was observed in the malformed cortex with peak increases near 10 Hz, a frequency dominated by SWDs in awake animals (Sun et al., 2016). In addition, the increases in spectral power extended into the high frequency range (>100 Hz) along the upper cortical layers including an increase in MUA. Clinical evidence from epileptic patients with cortical dysplasia indicates that a hyperexcitable zone exists within the dysplastic cortex adjacent to the microgyric lesion though it can extend into nearby structurally intact tissues (Mattia et al., 1995; Palmini et al., 1995; Guerrini and Dobyns, 2014). Similar *in vitro* results have also been obtained in several experimental studies that have defined electrophysiological hyperexcitabilities in both dysplastic cortex and adjacent regions of normatopic tissue (Jacobs et al., 1996; Luhmann et al., 1998; Redecker et al., 1998). In particular, a study by Redecker et al. (2005) indicated a defined region of epileptogenicity that was strongest in the upper layers of the dysplastic cortex which then spread to adjacent regions through superficial cortical layers as determined from optical imaging of neuronal activity during induced epileptiform events in cortical slice preparations. The specific anatomical or physiological alterations that drive the induced hyper-excitability surrounding the freeze-lesion induced microgyrus is currently unknown though altered expression patterns of excitatory and inhibitory receptors within this region has been established (Zilles et al., 1998; Redecker et al., 2000), likely leading to shifts in the normal excitatory/inhibitory tone of the affected tissue. Neonatal freeze-lesions also disrupt cortical cell function likely due to a hyper-innervation of ascending input into dysplastic regions that originally targeted cells within the microgyrus (Jacobs et al., 1999c; Rosen and Galaburda, 2000; Jacobs and Prince, 2005; Zhou and Roper, 2010). However, additional circuit analysis will be necessary to define the specific cortical circuits that may be responsible for the induced hyper-excitability observed in the supra-granular layer of the dysplastic cortex.

The current study also revealed a distinct pattern of altered LFP coherence in the malformed cortex offering further evidence of disturbed columnar and laminar communication network within the S1 region. The role of brain oscillations and their effect on neural communication is an active area of research that suggests that the synchrony or coherence of specific frequency bands are linked to specific behavioral and cognitive processes (Buzsáki and Schomburg, 2015). Specifically, a significant increase in LFP coherence was observed in freeze lesion animals across the supragranular layer in the upper gamma frequency range (50–200 Hz). In general, gamma-band synchronization is thought to be involved in the entrainment of neural networks (Singer and Gray, 1995; Jensen et al., 2007; Buzsáki and Silva, 2012) and may be a consequence of the increase in spectral activity as observed in the upper cortical layers in the current study. In contrast, a drop in LFP coherence between the upper and lower cortical layers was measured across beta and lower gamma frequencies (15–70 Hz). Disruption of beta rhythms within the S1 region may distort normal somatosensory processing (Fransen et al., 2016) and could lead to the observed behavioral changes that have been reported in this freeze lesion model (Sun et al., 2016).

Similar to the pattern of HFOs and increased spectral power observed across cortical lamina, spike sorting of spontaneous hyperexcitable unit activity in the malformed cortex was isolated to the upper half of the S1 region with a differential distribution of putative excitatory vs. inhibitory cells across layers. The majority of excitatory cells were recorded from the most superficial layers of the S1 cortex while inhibitory cells were mainly expressed in the deeper supragranular and granular layers. Although the highest incidence of HFO activity was observed in the most superficial cortical electrodes, the SFC of isolated single-units was strongest for deeper cortical layers, specifically for fast ripple frequencies ( >250 Hz). The differential distribution of cell types and stronger SFC values within the deep supragranular and granular layers has not been previously reported in FCD models or in patients with cortical dysplasia. Further analysis of excitatory cells recorded from the most superficial cortical layers indicated only weak phase-locking to HFOs in comparison to the strong phase-locking of inhibitory cells located in the lower supragranular layer to fast ripple band frequencies (possibly emanating from cells within the granular layer). These data suggest that the deeper inhibitory cells may be the driving substrate of ripple waves near the site of the microgyric lesion. It should be noted that these results were obtained under isoflurane anesthesia and should be verified in awake animals as well. However, in line with these results, the concept of abnormal glutamatergic excitation as the driving force behind initiation of epileptic discharges has been challenged in favor of a possible role of GABAergic inhibition in shaping ictal activity (de Curtis and Avoli, 2016). It should also be noted that the classification of SUAs into excitatory and inhibitory cells type is based on waveforms recorded in extracellular field potentials. A limitation of this method vs. intracellular recording is that the waveform varies base on a number of factors, including the composition of the electrode, impedance, distance from the recorded neuron, and distance to different cellular compartment in which the extracellular signals were generated, etc. Given the diversity of interneurons and their different firing properties, it has been estimated that the present methods may cause upward of 30% misclassified cell types for INH cells and about 5% neurons identified as EXC cell types might be interneurons (Reyes-Puerta et al., 2016). Despite this confounding factor, our data still indicate robust differences in phase coupling between INH vs. EXC cell types. However, we would like to caution readers to use caution when they use this portion of our results. A more robust relationship requires larger set of experimental data.

## Author Contributions

Q-QS designed the experiments, co-wrote the manuscript. AW designed the experiments, performed data analysis and wrote the manuscript.

## Conflict of Interest Statement

The authors declare that the research was conducted in the absence of any commercial or financial relationships that could be construed as a potential conflict of interest.
